# Proceedings of the Lippe Society

**Published:** 1889-08

**Authors:** George H. Clark

**Affiliations:** Secretary


### PROCEEDINGS OF THE LIPPE SOCIETY.

The 131st meeting of the Lippe Society was held on Tuesday evening, June 11th. Dr. C. Carleton Smith occupied the chair. After the minutes of the preceding meeting had been read and approved, Dr. M. Preston said he would like to have the Society turn aside from the regular order and discuss the significancy of nose-bleed in diphtheria. This was agreed to.

Dr. Preston—I wish to know the idea prevailing in respect of bleeding from the nose in diphtheria. In the past three weeks I have had four cases of diphtheria, one of which died in two days from the onset of the attack. In one case the membrane commenced by being blackish in the throat, and there was deep ulceration in the roof of the mouth and about the palate. The membrane also extended from the nose down over the lips, and was bleeding.

The one which died had no membrane in the throat, but there was a raw, ugly-looking ulcer, slightly pitted. It looked as though a polypus had been cut out, and resembled a cauliflower excrescence, though it was not above the surface. After looking through Gregg, I concluded that Bry. would have helped the case. Kali-bich. failed.

Dr. James—Speaking of Kali-bich., some time ago I had an old man over ninety years of age with erysipelas of one foot. I could find no remedy that would benefit him. After a short time a scab formed and there was a round, deep ulcer, as though it had been punched out, covered on the bottom with pus. Kali-bich. soon improved the case.

Dr. Preston—The significance of the symptom, nose-bleed, in diphtheria, is, in my opinion, a very serious, if not always one of fatal import. So far as my experience goes, when it occurs early in the diphtheritic onset it implies almost universally a fatal end in the case, particularly if the bleeding be several times renewed. The volume of blood discharged does not signify so much as the frequency of its occurrence. This frequent appearance of the bloody discharge would seem to indicate the existence of ulceration of more than commonly deep origin, the sloughing of the membrane from which would leave the superficial capillaries denuded and open.

The importance of hemorrhage, in a prognostic sense, has generally rested with those cases, so far as my observation extends, in which it has proceeded from the nostrils. From the cause I imagine that in the parts of the respiratory passages more secluded the ulcerative process is better protected, and hence less liable to give the early cry of alarm except where the disease may have assumed a more malignant aspect. In the pharynx we have a cavity which admits of easy and critical inspection, and hemorrhage thence is quite frequent in bad cases, but we are able to anticipate such a result and speak more intelligently of its purport when it occurs there. Hemorrhage has infrequently furnished the basis on which any medicine was selected, though we are not scantily supplied with remedies possessing such indications, yet they are, I believe, most generally called for on this account, but are corroborative merely.

The mental condition of the patient, the appearance and general conduct of the membranous deposit, and the subjective complaints, are, I believe, far the most valuable handles by which we can grasp this formidable destroyer. Hemorrhage corroboratively in the earliest stages, or mainly in the concluding phase of the disease, will constitute our best hold.

In all cases of diphtheria, even those that seem apparently mild, we must look out for danger-signals, for the changes from bad to worse are sometimes so remarkably rapid as to throw us entirely off our guard. There are three points of danger to be constantly kept in view, vix.: Extension of exudative process to the larynx, which is so rapidly fatal in children; the excessive exudation in the nares and nasal passages, and, lastly, hemorrhages from whatever portion of the body they may occur. Nasal hemorrhage or hemorrhage from kidneys I always consider a symptom of the utmost gravity, for it indicates decomposition of the blood, which, of course, means death, unless a speedy change is brought about by the carefully selected similar remedy.

By reason of their strong homœopathicity, we naturally look to the snake poisons for help in this condition of things, for the reason that they possess the peculiar power of producing just this condition of the blood when received into the circulation. And we consult, according to their symptoms, the Lachesis, the Elaps cor. fel., and the Vipera acuat. car., these being the leading members of the Aphidia.

Points: Bleeding persistent from nasal cavity, think of Elaps cor. fel.; bleeding with the blood thick and black, like treacle in cold weather, study Vipera acuat. car.; bleeding from roof of mouth as if dropping through a sieve slowly, Bryonia; a bloody sanious discharge, looking like blood and pus mixed, with wings of nose excoriated, think of Mercurius.

Delirium is another symptom which points to a fatal termination in this disease.

Abundance of albumen in the urine is not serious so long as the urine is normal in amount and without blood corpuscles or casts of tubes.

Dr. Clark—All authorities that I can recall speak of the gravity of the condition in which there is bleeding from the mucous surfaces. It is always considered of fatal import. That we need not feel so, our experience goes to confirm. Dr. Gregg, in his work on "Diphtheria," mentions a case in which the inner mouth, the tongue, and the parts of the fauces which could be seen discharged an acrid pus, which streamed from the mouth and from both nostrils. Bryonia$^{2000}$ was given, and the next day, instead of pus streaming out, the parts were all raw and bleeding, and blood was being discharged from the nose and chin. The Bry. was permitted to act, and the child made a good recovery.

GEORGE H. CLARK, *Secretary*.

